# Race/Ethnicity and the Pharmacogenetics of Reported Suicidality With Efavirenz Among Clinical Trials Participants

**DOI:** 10.1093/infdis/jix248

**Published:** 2017-07-06

**Authors:** Katie R Mollan, Camlin Tierney, Jacklyn N Hellwege, Joseph J Eron, Michael G Hudgens, Roy M Gulick, Richard Haubrich, Paul E Sax, Thomas B Campbell, Eric S Daar, Kevin R Robertson, Diana Ventura, Qing Ma, Digna R. Velez Edwards, David W Haas, and the AIDS Clinical Trials Group

**Affiliations:** 1 Center for AIDS Research and Departments of; 2 Neurology and; 3 Medicine, University of North Carolina at Chapel Hill;; 4 Center for Biostatistics in AIDS Research, Harvard T. H. Chan School of Public Health, and; 5 Division of Infectious Diseases and Department of Medicine, Brigham and Women’s Hospital and Harvard Medical School, Boston, Massachusetts;; 6 Division of Epidemiology, Department of Medicine, and; 7 Department of Obstetrics and Gynecology, Vanderbilt Genetics Institute, Vanderbilt University Medical Center,; 8 Department of Medicine, Vanderbilt University School of Medicine, and; 9 Department of Internal Medicine, Meharry Medical College, Nashville, Tennessee;; 10 Weill Cornell Medicine, Department of Medicine, New York, and; 11 University at Buffalo, Department of Pharmacy Practice, New York;; 12 Gilead Sciences, Foster City, and; 13 Los Angeles Biomedical Research Institute at Harbor–UCLA Medical Center, David Geffen School of Medicine at UCLA, California; and; 14 Department of Medicine, University of Colorado, Denver

**Keywords:** HIV, efavirenz, suicidality, pharmacogenetics, CYP2B6

## Abstract

**Background:**

We examined associations between suicidality and genotypes that predict plasma efavirenz exposure among AIDS Clinical Trials Group study participants in the United States.

**Methods:**

Four clinical trials randomly assigned treatment-naive participants to efavirenz-containing regimens; suicidality was defined as reported suicidal ideation or attempted or completed suicide. Genotypes that predict plasma efavirenz exposure were defined by *CYP2B6* and *CYP2A6* polymorphisms. Associations were evaluated with weighted Cox proportional hazards models stratified by race/ethnicity. Additional analyses adjusted for genetic ancestry and selected covariates.

**Results:**

Among 1833 participants, suicidality was documented in 41 in exposed analyses, and 34 in on-treatment analyses. In unadjusted analyses based on 12 genotype levels, suicidality increased per level in exposed (hazard ratio, 1.11; 95% confidence interval, .96–1.27) and on-treatment 1.16; 1.01–1.34) analyses. In the on-treatment analysis, the association was strongest among white but nearly null among black participants. Considering 3 metabolizer levels (extensive, intermediate and slow), slow metabolizers were at increased risk. Results were similar after baseline covariate-adjustment for genetic ancestry, sex, age, weight, injection drug use history, and psychiatric history or recent psychoactive medication.

**Conclusions:**

Genotypes that predict higher plasma efavirenz exposure were associated with increased risk of suicidality. Strength of association varied by race/ethnicity.

## Summary

This study examined associations between suicidality and genotypes that predict plasma efavirenz exposure among AIDS Clinical Trials Group study participants. Genotypes were associated with increased risk of suicidality. The association was strongest among white but nearly null among black participants.

Efavirenz is a frequently prescribed antiretroviral globally. Randomized clinical trials have demonstrated its efficacy [1–6]. However, some patients who start efavirenz experience central nervous system symptoms [7]. Among 5332 individuals who had been randomly assigned to start efavirenz-containing or efavirenz-free regimens in 4 AIDS Clinical Trials Group (ACTG) studies [8], efavirenz-containing regimens were associated with a 2-fold increased hazard of suicidality. In the START trial, patients who started efavirenz-containing regimens had a higher incidence of suicidality than those who deferred therapy, a difference not seen in those starting nonefavirenz regimens [9]. In contrast, no association with suicidality was apparent in the Food and Drug Administration Adverse Event Reporting System [10], or in United States administrative claims data for commercially and Medicaid-insured individuals [11]. In 2015, United States prescribing guidelines were updated to change efavirenz-containing regimens from recommended to alternative status as initial therapy for human immunodeficiency virus type 1 (HIV-1) infection [12].

Efavirenz is metabolized by cytochrome P450 (CYP) 2B6, with minor metabolism by CYP2A6 and CYP3A4/5 [13, 14], and direct N-glucuronidation by uridine diphosphate (UDP)-glucuronosyltransferase (UGT) 2B7 [15]. Several *CYP2B6* polymorphisms, including 516G→T (rs3745274) [16–21], 983T→C (rs28399499) [21–24], and 15582C→T (rs4803419), predict increased plasma efavirenz exposure [21]. The per allele effect is greatest with 983T→C and least with 15582C→T [21], such that combinations of the 3 polymorphisms predict 10 plasma concentration strata that span an approximately 10-fold range [21]. Slow metabolizer *CYP2B6* genotypes comprise the 3 highest strata, which are defined by either 516 T/T homozygosity, dual 516 G/T + 983 C/T heterozygosity, or 983 C/C homozygosity.

Polymorphisms beyond *CYP2B6,* particularly *CYP2A6*, also affect efavirenz pharmacokinetics [25, 26]. Plasma efavirenz concentrations are increased with *CYP2A6* -48T→G (rs28399433) [25–28], but only with concomitant *CYP2B6* slow metabolizer genotypes [25, 28]. A possible association has also been reported with *UGT2B7* genotype [27] although the effect size was small [28]. We examined whether genotypes known to predict increased plasma efavirenz exposure are associated with increased suicidality among individuals who were randomized to receive efavirenz-containing regimens in 4 ACTG studies.

## METHODS

### Study Design and Participants

The study design was similar to that of previous nongenetic analyses characterizing relationships between efavirenz and suicidality in these ACTG studies [8]. Data were pooled from antiretroviral-naive individuals who started therapy in 4 studies that had randomly assigned participants to efavirenz-containing versus efavirenz-free regimens: A5095 (ClinicalTrials.gov: NCT00013520) [2, 29], A5142 (NCT00050895) [3], A5175 (NCT00084136) [30], and A5202 (NCT00118898) [6]. Other than nucleoside analogue choice in A5142, drug class components of the regimens were randomly assigned. The present analyses were also restricted to self-reported white, black, and Hispanic participants (classified according to National Institutes of Health categories) at ACTG sites in the United States (including Puerto Rico). Genetic testing was restricted to participants who consented under ACTG protocol A5128 [31]. Other race/ethnicity groups (eg, Asian) included too few individuals for analysis. Primary analyses were conducted among individuals assigned to efavirenz-containing regimens (hereafter called the efavirenz group), and a supplemental analysis included those assigned to efavirenz-free regimens for comparison (hereafter called the nonefavirenz group).

History of suicidal ideation or attempt was not exclusionary. Protocols required reporting of signs, symptoms, or diagnoses at each visit, which were recorded with open-text and data-entry codes. Each study required reporting of severe and life-threatening graded signs or symptoms per the Division of AIDS grading table [32], as well as signs or symptoms that led to change in study treatment. Diagnoses were not graded. Further, study A5142 required report of all moderate signs or symptoms, and A5095 and A5202 required report of moderate central nervous system symptoms. Site institutional review boards approved each study, and participants provided written informed consent.

### Outcomes

The primary outcome was suicidality, defined as suicidal ideation or attempted or completed suicide and identified from signs, symptoms, diagnoses, adverse events, and death data as described in detail elsewhere [8]. Attempted or completed suicide was a secondary outcome, as was suicidality or fatal injury attributed to substance abuse, homicide, accident, or unknown cause. The at-risk period started when antiretroviral treatment was initiated. For efavirenz-exposed analyses, subsequent follow-up was included regardless of treatment status. For on-treatment analyses, follow-up was censored at discontinuation of the randomly assigned efavirenz strategy, plus 28 days for washout.

### Covariates

Baseline covariates included for adjustment were self-reported race/ethnicity (or, alternatively, genetic ancestry), sex, age category, history of injection drug use, documented psychiatric history or use of psychoactive medication within 30 days before study entry, and body weight category. CD4 T-cell count, HIV-1 RNA, and history of AIDS-defining event were also measured at baseline. Genome-wide genotype data were used to generate principal components that were also used as covariates to adjust for genetic ancestry, to minimize confounding by unrecognized population stratification (see Statistical Analysis section for details).

### Genetic Assays and Data

Genotypes for *CYP2B6* 516G→T, 983T→C, 15582C→T and *CYP2A6* -48T→G were largely available from a MassARRAY iPLEX Gold (Sequenom) assay, generated by Vanderbilt Technologies for Advanced Genomics (VANTAGE) as described elsewhere [21]. For additional participants who consented to genetic testing but lacked such data, *CYP2B6/CYP2A6* genotyping was attempted with the same assay. Genome-wide genotype data largely available from a previous immunogenomics project [33] were generated by Illumina HumanHap 650Y array for A5095 and by Illumina 1M duo array for A5142 and A5202. Quality control and imputation of genome-wide data were performed essentially as described elsewhere [34]. To assure that genotypes could be combined across assays, 65 samples previously genotyped with HumanHap 650Y array or Illumina 1M duo array were regenotyped with Illumina Expanded Multi-Ethnic Genotyping array (MEGAEX, with >2 million markers). There was high concordance across genotypes (r^2^ > 0.998) and the first 10 principal components (all r^2^ > 0.998), thus providing assurance. In addition, MEGAEX genotyping was attempted in participants with suicidality in whom genome-wide genotype data were not already available.

Efavirenz metabolizer categories were prespecified, and were defined by combinations of *CYP2B6* and *CYP2A6* polymorphisms [21, 28]. Two categorization approaches considered the full range metabolizer strata based on either *CYP2B6* alone (10 strata) or both *CYP2B6* and *CYP2A6* (12 strata). A third approach collapsed the 12 strata into 3 groups, defined as extensive metabolizers (strata 1–2), intermediate metabolizers (strata 3–7), and slow metabolizers (strata 8 and higher). See **Supplemental Material** for details. Consent for genetic analysis was obtained under ACTG protocol A5128 [31], and the ACTG approved this use of DNA.

### Statistical Analysis

Our aim was to estimate the association between efavirenz metabolizer genotype and suicidality among participants who started randomly assigned efavirenz-containing regimens for initial treatment of HIV-1 infection. The association between genotype level and time to suicidality outcomes was evaluated with a hazard ratio (HR) from a weighted Cox proportional hazards model, stratified by race/ethnicity group, with genotype level included as a linear covariate. To assess the linearity assumption, genotype level was also fit using a restricted quadratic spline with 4 equally spaced knots at the 20th, 40th, 60th and 80th percentiles [35]. Furthermore, metabolizer genotype was fit as 3 unordered groups versus linear with 3 levels (extensive, intermediate, slow); a likelihood ratio test did not provide evidence against the linear fit (*P* = .49 for efavirenz-exposed and *P* = .93 for efavirenz on-treatment analysis). Subsequent analyses include genotype as a linear covariate unless indicated otherwise.

Further analyses were conducted to adjust for genetic ancestry among the subset of participants with genome-wide genotype data. To quantify ancestry among samples, we estimated continuous axes of ancestry incorporating the intersection of common autosomal genotypes across all genotyped sets used (n = 74308 single-nucleotide polymorphisms) using the program EIGENSTRAT version 6.1.4 [36]. Samples from the International HapMap Project [37] phase 3 were also included to provide global reference populations. The principal components scree plots were visually examined to ensure that the components selected for analyses represented ancestral information. The first 4 genetic ancestry principal components were included as covariates in a weighted Cox model that was not stratified by self-reported race/ethnicity; additional principal components were not included to avoid model overfitting. Adjusted analyses included additional baseline covariates as described above. Race/ethnicity (and genetic ancestry) was the primary confounding factor and potential modifier of interest, and small event numbers limited the number of additional covariates in the adjustment set for multivariable analyses.

A supplemental analysis evaluated whether participants classified as efavirenz extensive metabolizers in the efavirenz group were at greater risk of suicidality than those who were randomly assigned to the nonefavirenz group. As with the efavirenz group, the nonefavirenz group was restricted to white, black, and Hispanic participants who enrolled in the United States or Puerto Rico and who consented to genetic testing. *CYP2B6/CYP2A6* testing was not conducted in the nonefavirenz group. A 2-sided 0.05 statistical significance level was used, with no adjustment for multiple testing. Statistical analyses were conducted using Windows SAS (version 9.4) and R (version 3.3.1) software.

### Weighting for Missing Data and Dropout

The 2239 participants who started randomly assigned efavirenz therapy were considered the full sample. Inverse probability missingness weights were used to adjust for 18% missing metabolizer genotype data and 38% missing genetic ancestry data [38]. Inverse probability censoring weights were used in Cox models to adjust for event-free dropout due to (1) premature study discontinuation for the exposed follow-up analyses and (2) premature discontinuation of the assigned efavirenz strategy or study follow-up for on-treatment analyses [39]. For the exposed follow-up analyses, using inverse probability weights, we aimed to estimate the association between genotype level and suicidality in a setting where all participants who started efavirenz therapy had genetic data available and also completed study follow-up. For the on-treatment analyses, we aimed to estimate this same association in a setting where participants who started efavirenz therapy had genetic data available and remained on the efavirenz regimen and in follow-up for their full possible duration of study. Participants who could not continue owing to death or research site defunding were considered study completers.

Logistic regression was used to estimate missingness weights, and pooled logistic regression to estimate censoring weights. The product of these 2 weights was applied in a weighted Cox model using the robust variance estimator [40]. Five fully observed baseline covariates were included to estimate weights: sex, self-reported race/ethnicity, age (restricted quadratic spline), history of injection drug use, and psychiatric history or psychoactive medication. The missingness weights model included these 5 covariates plus an indicator of suicidality outcome to account for differential data availability; the censoring weights model included these 5 covariates as well as *CYP2B6/CYP2A6* metabolizer level. Main effects and 2-way interactions (linear interactions for age) were included in the models to estimate inverse probability of missingness and censoring weights.

## RESULTS

### Study Participants

**Figure 1.**
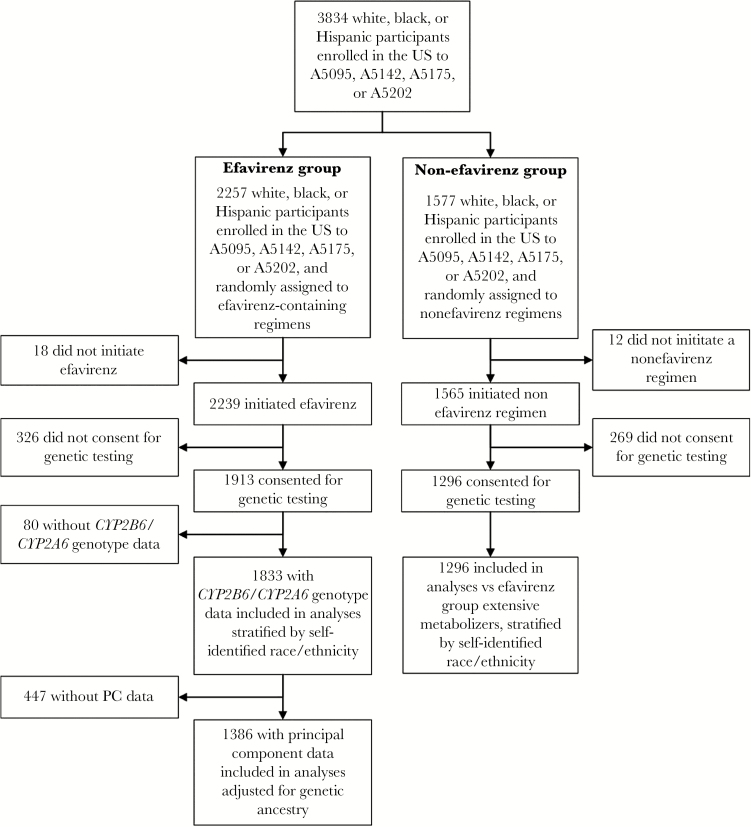
Derivation of the study sample. Selection of the study population is shown among white, black, and Hispanic participants who were randomly assigned to an efavirenz-containing or nonefavirenz regimen in A5095, A5142, A5175, or A5202 and enrolled in the United States (US) or Puerto Rico. PC, principal component.

A total of 2257 white, black, or Hispanic participants from ACTG studies A5095, A5142, A5175, and A5202 were randomly assigned to receive efavirenz-containing regimens at research sites in the United States and Puerto Rico, of whom 2239 started efavirenz therapy. After exclusion of those who did not provide consent for genetic testing or were not successfully genotyped for *CYP2B6* and *CYP2A6*, 1833 (82% of 2239) participants were evaluable for associations between *CYP2B6*/*CYP2A6* genotype and suicidality. These included 41 (87% of 47) with suicidality and 1792 (82% of 2192) without suicidality. Of these 1833 participants, 1386 also had ancestry principal components data, including 40 (85% of 47) with suicidality and 1346 (61% of 2192) without suicidality, the latter lower percentage reflecting our decision to generate ancestry principal components in as many participants with suicidality as possible, while relying on previously available genotype data to generate principal components on those without suicidality. Participant disposition is presented in Figure 1.

Baseline characteristics and genotype frequencies are shown in Table 1. Among the 1833 participants with *CYP2B6*/*CYP2A6* genotype data, the median age was 38 years, and 18% were female, generally consistent with the remaining 406 individuals who did not have *CYP2B6*/*CYP2A6* genotype data, except that those with *CYP2B6*/*CYP2A6* genotype data were more likely to be white. Genotype frequencies in each race/ethnicity group are presented in Table 2. Slow metabolizer genotypes were present in 50 (6%) of 781 white, 120 (18%) of 660 black, and 45 (11%) of 392 Hispanic participants.

**Table 1.** Baseline Characteristics of Study Participants Assigned to an Efavirenz-Containing Regimen

Characteristic	*CYP2B6/CYP2A6* Genotype Data Available, No. (%)^a^		Overall, No. (%)^a^ (n = 2239)
	Yes (n = 1833)	No (n = 406)	
Parent study			
A5095	635 (35)	105 (26)	740 (33)
A5142	403 (22)	70 (17)	473 (21)
A5175	104 (6)	32 (8)	136 (6)
A5202	691 (38)	199 (49)	890 (40)
Sex			
Male	1510 (82)	316 (78)	1826 (82)
Female	323 (18)	90 (22)	413 (18)
Race/ethnicity			
White non-Hispanic	781 (43)	121 (30)	902 (40)
Black non-Hispanic	660 (36)	166 (41)	826 (37)
Hispanic	392 (21)	119 (29)	511 (23)
Age, y			
Median (IQR)	38 (31–44)	38 (30–44)	38 (31–44)
Range	17–77	19–71	17–77
CD4 T-cell count, cells/μL^b^			
Median (IQR)	218 (75–330)	188 (64–295)	212 (73–322)
Range	0–1336	1–849	0–1336
HIV-1 RNA, log_10_ copies/mL			
Median (IQR)	4.74 (4.39–5.24)	4.74 (4.40–5.22)	4.74 (4.39–5.24)
Range	2.34–7.04	2.76–6.71	2.34–7.04
History of AIDS	326 (18)	63 (16)	389 (17)
History of injection drug use	174 (9)	49 (12)	223 (10)
Psychiatric history or psychoactive medication	711 (39)	148 (36)	859 (38)
Psychoactive medication	310 (17)	69 (17)	379 (17)
Depression-related history or antidepressant medication	459 (25)	94 (23)	553 (25)
Antidepressant medication	239 (13)	53 (13)	292 (13)
Body mass index (kg/m^2^)^c^			
Median (IQR)	24.6 (22.0–27.8)	24.9 (22.2–27.9)	24.7 (22.1–27.8)
Range	13.9–60.6	17.1–53.8	13.9–60.6

Abbreviations: HIV-1, human immunodeficiency virus type 1; IQR, interquartile range.

^a^Data represent No. (%) of participants unless otherwise specified.

^b^This analysis included 2237 participants (1831 with and 406 without *CYP2B6/CYP2A6* genotype data available).

^c^This analysis included 2205 participants (1810 with and 395 without *CYP2B6/CYP2A6* genotype data available).

**Table 2.** Genotype Frequencies According to Self-Identified Race/Ethnicity

Metabolizer Level	*CYP2B6*			*CYP2A6*: -48T→G	White (n = 781)	Black (n = 660)	Hispanic (n = 392)	Overall (n = 1833)
	15582C→T	516G→T	983T→C					
Extensive					*355 (45)*	*218 (33)*	*115 (29)*	*688 (38)*
1	CC	GG	TT	-	166 (21)	137 (21)	41 (10)	344 (19)
2	CT	GG	TT	-	189 (24)	81 (12)	74 (19)	344 (19)
Intermediate					*376 (48)*	*322 (49)*	*232 (59)*	*930 (51)*
3	TT	GG	TT	-	94 (12)	12 (2)	68 (17)	174 (9)
4	CC	GT	TT	-	176 (23)	198 (30)	69 (18)	443 (24)
5	CC	GG	CT	-	0 (0)	45 (7)	1 (0)	46 (3)
6	CT	GT	TT	-	106 (14)	57 (9)	93 (24)	256 (14)
7	CT	GG	CT	-	0 (0)	10 (2)	1 (0)	11 (1)
Slow					*50 (6)*	*120 (18)*	*45 (11)*	*215 (12)*
8	CC	TT	TT	-	47 (6)	81 (12)	40 (10)	168 (9)
9	CC	GT	CT	-	0 (0)	15 (2)	2 (1)	17 (1)
10	CC	GG	CC	-	0 (0)	2 (0)	0 (0)	2 (0)
11			Level 8, 9, or 10	GT	2 (0)	22 (3)	3 (1)	27 (1)
12			Level 8, 9, or 10	GG	1 (0)	0 (0)	0 (0)	1 (0)
Total								

Data are no. (%).

### Associations With Suicidality

Among the 1833 participants with *CYP2B6*/*CYP2A6* genotype data, suicidality was documented in 41 (2.2%) over 4743 person-years at risk in the efavirenz-exposed analysis (estimated incidence, 8.64/1000 person-years), and 34 (1.9%) over 3954 person-years at risk in the on-treatment analysis (8.60/1000 person-years). In unadjusted weighted analyses based on 12 genotype levels defined by *CYP2B6*/*CYP2A6* genotypes (our most precise genetic estimate of plasma efavirenz exposure), the estimated hazard of suicidality was increased per level in both exposed (HR, 1.11; 95% confidence interval [CI], .96–1.27; *P* = .16) and on-treatment (1.16; 1.01–1.34; *P* = .04) analyses, although only the latter was statistically significant. 

**Figure 2.**
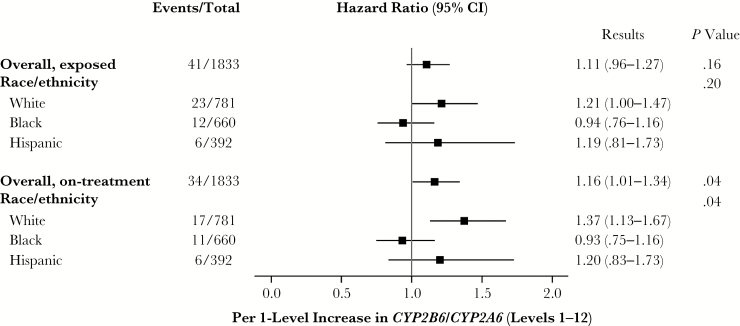
Relative hazard of suicidality by genotype level among participants randomly assigned to efavirenz-containing regimens. Each incremental *CYP2B6*/*CYP2A6* genotype level (levels 1–12) is known to be associated with progressively greater plasma efavirenz exposure. The estimated relative hazard of suicidality is shown overall and within each race/ethnicity group, for both exposed and on-treatment risk periods. Overall *P* values test reflect the main effect of genotype level; race/ethnicity *P* values, a statistical interaction between genotype level and race/ethnicity group. Hazard ratios were estimated from a weighted Cox model stratified by race/ethnicity; robust Wald confidence intervals (CIs) and *P* values are shown.

**Figure 3.**
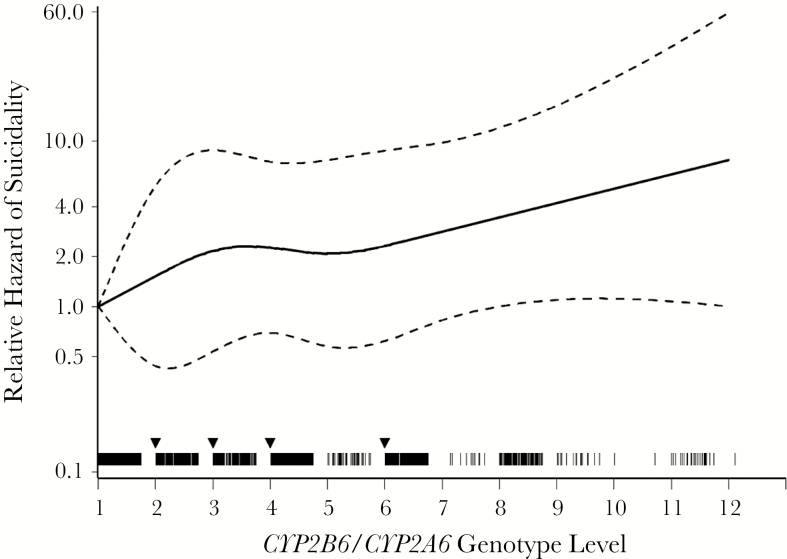
Genotype level fit with a quadratic spline. *CYP2B6/CYP2A6* genotype level was fit with a quadratic spline with 4 equally spaced knots at levels 2, 3, 4, and 6, as indicated by downward arrows. Each hash mark represents the participant’s observed genotype level (n = 1833); solid line, the estimated relative hazard; and dashed lines, pointwise 95% Wald-type confidence intervals from a weighted Cox model stratified by race/ethnicity group. This provides support for the assumption of a linear association with no apparent threshold effect. The unweighted result was similar and slightly closer to linear (data not shown).

There was significant effect measure modification by race/ethnicity in the on-treatment analysis, with the positive association between genotype level and suicidality being strongest among white, intermediate among Hispanic, and not present among black participants (*P* = .04). In the exposed analysis the pattern was similar, although the association was partially attenuated among white participants, and significant effect measure modification was not detected (*P* = .20) (Figure 2). To assess the assumption of linearity of the 12 genotype levels (which predict progressively higher plasma efavirenz exposure) and suicidality, genotype level was also fit using a restricted quadratic spline. Visual inspection of the spline fit provided support for the assumption of a linear association with no apparent threshold effect (Figure 3).

**Figure 4.**
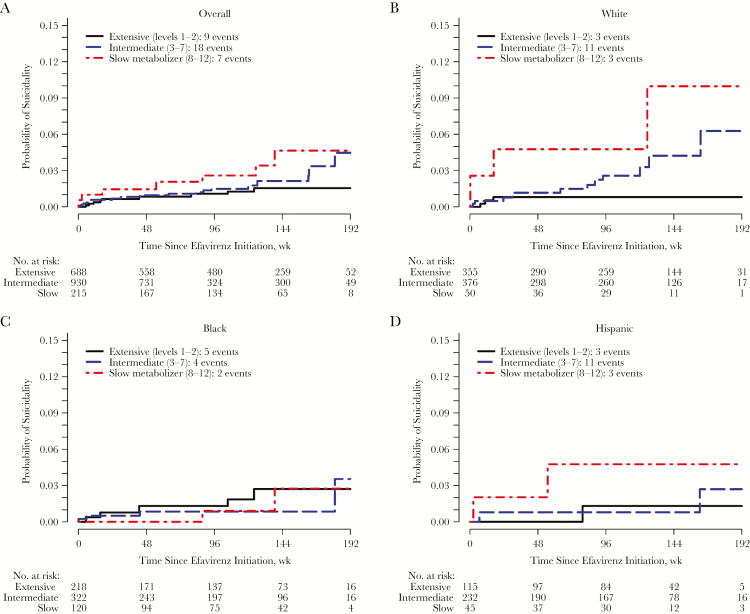
Cumulative probability of suicidality for each metabolizer group in on-treatment analysis, overall and by race/ethnicity. *A,* Estimated cumulative probability of suicidality for each metabolizer group is shown overall among all participants (*A*) and among white (*B*), black (*C*), and Hispanic (*D*) participants. Values for probability represent 1 minus the weighted Kaplan-Meier estimate; on-treatment analyses are shown. The unweighted number at risk is shown below each panel, and the number of suicidality events is provided in the key.

The estimated cumulative probability of suicidality based on 3 genotype levels is presented in Figure 4. Among all study participants, slow metabolizers were at numerically increased risk at each time point, with no apparent difference between intermediate and slow metabolizers during most of the study follow-up period (Figure 4A). This differed by race/ethnicity. Among black participants, neither slow nor intermediate metabolizers seemed to be at greater risk than extensive metabolizers, whereas among white and Hispanic participants, there seemed to be an association between metabolizer level and suicidality, although there were relatively few Hispanic participants (Figure 4B–4D). Table 3 presents results from weighted Cox models to estimate the association between genotype level and a suicidality outcome adjusted for self-identified race/ancestry, as well as for psychiatric history or psychoactive medication, injection drug use history, sex, age category, and body weight category.

**Table 3.** Associations Between Genotype level and Suicidality, Adjusted for Genetic Ancestry and Other Covariates^a^

Analysis Period, Adjustment, and Efavirenz Metabolizer Measure	Estimated HR (95% CI)	Confidence Limit Ratio (Precision)	Wald *P* value
Efavirenz-exposed analysis			
Self-identified race/ethnicity (n = 1831; 41 events)			
10 level	1.12 (.97–1.29)	1.3	.13
12 level	1.11 (.97–1.28)	1.3	.12
3 level	1.48 (.85–2.56)	3.0	.17
Principal components (n = 1384; 40 events)			
10 level	1.10 (.96–1.27)	1.3	.17
12 level	1.08 (.96– 1.23)	1.3	.21
3 level	1.42 (.84– 2.41)	2.9	.19
On-treatment analysis			
Self-identified race/ethnicity (n = 1831; 34 events)			
10 level	1.17 (1.01–1.36)	1.3	.04
12 level	1.16 (1.01–1.33)	1.3	.04
3 level	1.87 (1.05–3.31)	3.2	.03
Principal components (n = 1384; 33 events)			
10 level	1.15 (.99–1.32)	1.3	.06
12 level	1.12 (.99–1.27)	1.3	.08
3 level	1.74 (1.02–2.97)	2.9	.04

Abbreviations: CI, confidence interval; HR, hazard ratio.

^a^Each weighted Cox model to estimate the association between genotype level and a suicidality outcome was adjusted either for self-identified race/ethnicity or for genetic ancestry using 4 principal components. Each analysis also adjusted for psychiatric history or psychoactive medication, injection drug use history, sex, age category, and body weight category. In the multivariable adjusted analyses the events to covariates ratio is low, with 33–41 events, and covariates using 12 degrees of freedom. Results adjusted only for race/ethnicity or principal components 1–4 were similar.

The above analyses were stratified by self-identified race/ethnicity, which can introduce unrecognized population stratification. Additional adjusted analyses controlled for genetic ancestry based on the first 4 principal components, as well as psychiatric history/psychoactive medication, history of injection drug use, sex, age category, and body weight category. Among 1384 participants (including white, black, and Hispanic participants) with both principal component and other covariate data, the HR estimate by genotype level for suicidality exceeded 1.0 in all analyses but was statistically significant only in on-treatment analysis based on 3 metabolizer levels (HR, 1.74; 95% CI, 1.02–2.97; *P* = .04). Results from weighted Cox models to estimate the association between genotype level and a suicidality outcome adjusted for the first 4 principal components, as well as for psychiatric history or psychoactive medication, injection drug use history, sex, age category, and body weight category, are presented in Table 3. Results of weighted Cox models were similar in analyses that considered not only suicidality but also death from substance abuse, accident, or unknown cause (data not shown).

### Comparison to Efavirenz-Free Regimens

We explored risk of suicidality among *CYP2B6*/*CYP2A6* extensive metabolizers versus individuals randomized to nonefavirenz regimens in these clinical trials, and who also consented to genetic testing. There were 12 (0.9%) suicidality events reported among 1296 individuals in the nonefavirenz group. In on-treatment analyses, the hazard of suicidality was numerically lower in the nonefavirenz group than in the extensive metabolizer group, although this difference was not statistically significant (HR, 0.70; 95% CI, .29–1.67; *P* = .42). The estimated probability of suicidality in the nonefavirenz group was numerically lower than in the extensive metabolizer group at every time point. Among white participants, the likelihood of suicidality was similar in the extensive metabolizer and nonefavirenz groups. Among black participants, the number with suicidality was small in both the extensive metabolizer and nonefavirenz groups. Cumulative probabilities of suicidality in relation to the nonefavirenz group are in the **Supplemental Material**.

## DISCUSSION

In ACTG studies, randomization to initial treatment with efavirenz-containing regimens was previously reported to be associated with a 2-fold increased hazard of suicidality [8]. The present study showed that, in analyses limited to white, black, and Hispanic participants randomly assigned to receive an efavirenz-containing regimen in those same studies, genotypes that predict increased plasma efavirenz exposure were also associated with increased suicidality. This finding further supports the validity of the reported association between efavirenz and suicidality [8]. In addition, the genetic association with suicidality progressively increased by *CYP2B6*/*CYP2A6* genotypes that predict progressively higher plasma efavirenz concentrations with no apparent threshold, suggesting that increased suicidality is not limited to *CYP2B6* slow metabolizers. Our study also showed an apparent difference in the on-efavirenz treatment association between genetics and suicidality depending on race/ethnicity, with an association among white participants (HR. 1.37; 95% CI, 1.13–1.67) but not black participants (0.93; .75–1.16). This analysis included fewer Hispanic participants, but their risk seemed to be intermediate (HR, 1.20; 95% CI, .83–1.73).

This study replicates a difference by race first noted in a much smaller ACTG data set [24] in which there was an association between *CYP2B6* slow metabolizer genotypes and increased central nervous system adverse events in 276 white participants (*P* = .04) but not in 217 black participants (*P* = .58). A subsequent observational study involving 563 patients who started efavirenz-containing regimens at a clinic in the southeastern United States found that slow metabolizer *CYP2B6* genotypes were associated with efavirenz discontinuation for reported central nervous system symptoms in 335 white participants (*P* = .001) but not in 198 black participants (*P* = .27) [41]. These previous studies, together with the present study, suggest that among patients with intermediate or slow metabolizer genotypes who are prescribed efavirenz in the United States, central nervous system side effects, including suicidality, are more likely to be reported among white than among black participants. Among Hispanic participants, *CYP2B6*/*CYP2A6* genotype level also seemed to be associated with increased suicidality, although this association was not statistically significant and the confidence interval was wide, reflecting the small number of events in these analyses.

We cannot explain the stronger association of *CYP2B6*/*CYP2A6* slow metabolizer genotypes with suicidality among white compared with black participants. Black participants with suicidality may have been less likely to report such symptoms than white participants with similar symptoms, resulting in misclassification. Plasma efavirenz exposure is similar among black and white individuals with *CYP2B6* slow metabolizer genotypes [21], so differences in such exposure are unlikely to explain the attenuated genetic association in blacks compared with whites. It is also possible that polymorphisms in genes beyond *CYP2B6* affect susceptibility to suicidality with efavirenz.

Genetic testing has the potential to identify patients in whom drugs with unfavorable adverse event profiles may be safely prescribed. This was the rationale for exploring whether extensive metabolizers (ie, lower-risk subgroup) in the efavirenz group were still at greater risk of suicidality than participants in the nonefavirenz group. The numerically lower probability of suicidality in the nonefavirenz group at each study time point overall supports the validity of the association between *CYP2B6* genotype level and suicidality in the efavirenz group. Although among white participants the likelihood of suicidality was similar in the extensive metabolizer and nonefavirenz groups, the small number of participants with suicidality in both groups prevents us from concluding that *CYP2B6* genotyping can identify patients at no increased risk of suicidality with efavirenz versus nonefavirenz regimens.

Unrecognized population stratification can cause spurious associations in genetic epidemiology studies. We addressed this by not only considering self-identified race/ethnicity but by also separately adjusting for principal components generated from genome-wide genotype data. Associations persisted in the latter analyses. In addition, the genetic association persisted in multivariable models that also included sex, age category, history of injection drug use, psychiatric history or psychoactive medication, and body weight category (all measured at baseline).

Our study had limitations. Because data were from clinical trials that did not specifically focus on suicidality, we may have missed some suicidality cases. To help address the possibility that suicidality was underreported, we included suicidality plus death from substance abuse, accident, or unknown cause in sensitivity analyses. Because providers may not have referred participants considered to be at high risk for neurologic intolerance to studies with efavirenz-containing regimens, risk of suicidality may be underestimated. Analyses were limited to white, black, and Hispanic participants at sites in the United States, so findings may not translate to other countries or race/ethnicities, including Asians. The open-label design of 3 of the 4 studies might have biased investigators into reporting neuropsychiatric effects in patients randomized to efavirenz.

In summary, *CYP2B6*/*CYP2A6* genotypes were associated with increased reported suicidality among participants who had been randomly assigned to receive efavirenz-containing regimens in clinical trials. This association was most apparent in white participants and attenuated in black participants.

## Supplementary Data

Supplementary materials are available at *The Journal of Infectious Diseases* online. Consisting of data provided by the authors to benefit the reader, the posted materials are not copyedited and are the sole responsibility of the authors, so questions or comments should be addressed to the corresponding author.

## Supplementary Material

Supplementary_Material

## Notes

***Acknowledgments.*** We are grateful to the many persons with HIV infection who volunteered for A5095, A5142, A5175, A5202, and A5128. We also acknowledge the contributions of study teams and site staff for these protocols.

***Financial support.*** This work was supported by the National Institute of Allergy and Infectious Diseases (NIAID) (grant U01AI068636); the National Institute of Mental Health; National Institute of Dental and Craniofacial Research; the ACTG, funded by the NIAID (grants AI-068636, AI-038858, AI-068634, and AI-038855); (NIAID grants AI-069439 and National Center for Translational Sciences (NCATS) TR-000445 to D. W. H.); the University of North Carolina at Chapel Hill Center for AIDS Research (grant P30 AI50410); and the Vanderbilt Molecular and Genetic Epidemiology of Cancer training program, funded by the US National Cancer Institute (grant R25 CA-160056; support to J. N. H.). Clinical research sites that participated in ACTG protocols A5095, A5142, A5175 and A5202 and collected DNA under protocol A5128 were supported by NIAID (grants AI069477, AI027675, AI073961, AI069474, AI069432, AI069513, AI069423, AI050410, AI069452, AI69450, AI054907, AI069428, AI069439, AI069467, AI045008, AI069495, AI069415, AI069556, AI069484, AI069424, AI069532, AI069419, AI069471, AI025859, AI069418, AI050409, AI069423, AI069501, AI069502, AI069511, AI069434, AI069465, AI069471, AI069494, AI069424, AI069472, AI069501, AI069470, AI046376, AI069511, AI072626, AI069472, AI038858, AI069472, AI027661, AI034853, AI069447, AI032782, AI027658, AI27666, AI058740, and AI046370) and by the National Center for Research Resources (grants RR00051, RR00046, RR025747, RR025777, RR024160, RR024996, and RR024156). Abbott Laboratories, Boehringer-Ingelheim, Bristol-Myers Squibb, Gilead Sciences, and GlaxoSmithKline provided study medications.

***Potential conflicts of interest.*** K. R. M. has received research support from grants awarded to the University of North Carolina at Chapel Hill from Merck, AbbVie, and Gilead. J. J. E. has received research support from grants awarded to the University of North Carolina at Chapel Hill from Bristol-Myers Squibb (BMS), AbbVie, Janssen, ViiV Healthcare, and Gilead and is a consultant to BMS, AbbVie, Janssen, ViiV Healthcare, Merck, and Gilead. R. H. is an employee of Gilead Sciences. P. E. S. has received research support from grants awarded to Brigham and Women’s Hospital from BMS, ViiV, and Gilead and is a consultant to BMS, AbbVie, Janssen, ViiV, Merck, and Gilead. T. B. C. has served as a consultant to Gilead Sciences. E. S. D. has received research support from grants awarded to Los Angeles Biomedical Research Institute at Harbor-UCLA Medical Center from Gilead, Merck, and ViiV and is a consultant for BMS, Janssen, Merck, Teva, and ViiV. Q. M. has received research support from grants awarded to the University at Buffalo from Gilead. All other authors report no potential conflicts. All authors have submitted the ICMJE Form for Disclosure of Potential Conflicts of Interest. Conflicts that the editors consider relevant to the content of the manuscript have been disclosed.
