# Supplementary material for: Race/Ethnicity and the Pharmacogenetics of Reported Suicidality With Efavirenz Among Clinical Trials Participants
Source: J Infect Dis. 2017 Jul 6;216(5):554–64. doi: 10.1093/infdis/jix248 (PMC5853681; doi:10.1093/infdis/jix248)

Supplemental Table: Definition of *CYP2B6* and *CYP2B6*/*CYP2A6* efavirenz metabolism genotype levels. CYPCOMP3 indicates combinations of 10- or 12-level metabolism genotype levels into 3-level metabolism genotype levels.

CYP2B6_met	rs28399433	rs4803419	rs3745274	rs28399499	CYPCOMP3	EFV_level
1	-	C C	G G	T T	Extensive	lowest
2	-	C T	G G	T T	Extensive	
3	-	T T	G G	T T	Mid	
4	-	C C	G T	T T	Mid	
5	-	C C	G G	C T	Mid	
6	-	C T	G T	T T	Mid	
7	-	C T	G G	C T	Mid	
8	-	C C or CT	T T	T T	Slow	
9	-	C C	G T	C T	Slow	
10	-	C C	G G	C C	Slow	highest

CYP2A6_hi	rs28399433	rs4803419	rs3745274	rs28399499	CYPCOMP3	EFV_level
1	-	C C	G G	T T	Extensive	lowest
2	-	C T	G G	T T	Extensive	
3	-	T T	G G	T T	Mid	
4	-	C C	G T	T T	Mid	
5	-	C C	G G	C T	Mid	
6	-	C T	G T	T T	Mid	
7	-	C T	G G	C T	Mid	
8	-	C C or CT	T T	T T	Slow	
9	-	C C	G T	C T	Slow	
10	-	C C	G G	C C	Slow	
11	G T	C C	T T	T T	Slow	
11	G T	C C	G T	C T	Slow	
11	G T	C C	G G	C C	Slow	
12	G G	C C or CT	T T	T T	Slow	
12	G G	C C	G T	C T	Slow	
12	G G	C C	G G	C C	Slow	highest

Supplemental Figure 1. Cumulative probability of suicidality for each metabolizer group among participants who were randomly assigned to efavirenz-containing regimens, and in the efavirenz-free group regardless of metabolizer group, overall and by race/ethnicity. *Panel A:* Estimated cumulative probability of suicidality for each metabolizer group overall among all participants. *Panel B:* Cumulative probability of suicidality for each metabolizer group White participants. *Panel C:* Cumulative probability of suicidality for each metabolizer group among Black participants. *Panel D:* Cumulative probability of suicidality for each metabolizer group among Hispanic participants. One minus the Kaplan-Meier estimate is shown. The number at risk is shown below each panel and the number of suicidality events is provided in the legend.

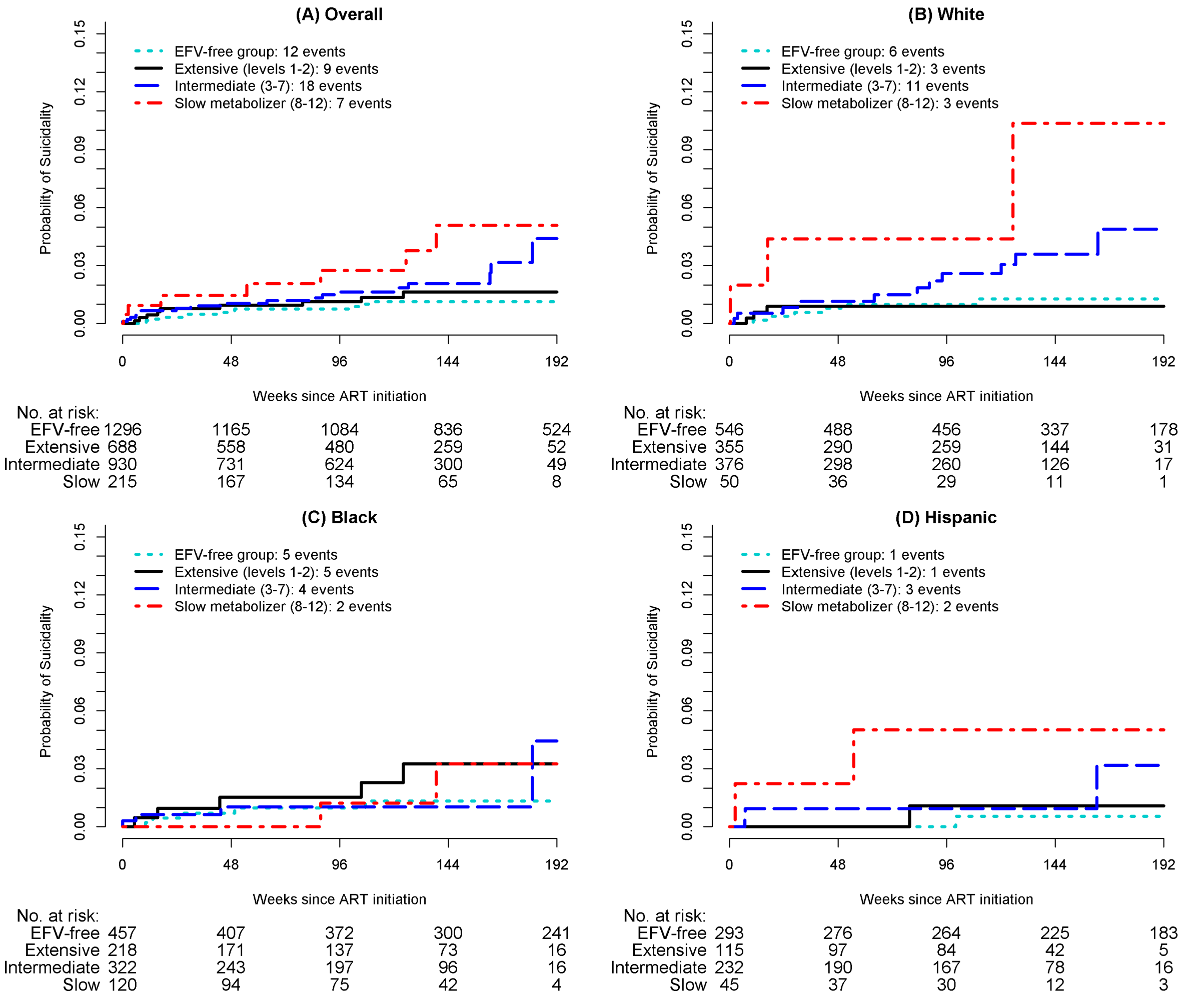

Supplement: Supplementary_Material [file jix248_suppl_supplementary_material.pdf]
